# Synthesis, Characterization, and Mechanism of Formation of Janus-Like Nanoparticles of Tantalum Silicide-Silicon (TaSi_2_/Si)

**DOI:** 10.3390/nano5010026

**Published:** 2014-12-25

**Authors:** Andrey V. Nomoev, Sergey P. Bardakhanov, Makoto Schreiber, Dashima Zh. Bazarova, Boris B. Baldanov, Nikolai A. Romanov

**Affiliations:** 1 Institute of Physical Materials Science, Siberian Branch of the Russian Academy of Sciences, Sakhyanovoy str., 6, Ulan-Ude 670047, Russia; 2 Department of Physics and Engineering, Buryat State University, Smolina str., 24a, Ulan-Ude 670000, Russia; E-Mails: bardnski@gmail.com (S.P.B.); mschreib@mail.uoguelph.ca (M.S.); ars-d@mail.ru (D.Z.B.); boris.baldanov@mail.ru (B.B.B.); nromanovv@mail.ru (N.A.R.); 3 Institute of Applied and Theoretical Mechanics, Siberian Branch of the Russian Academy of Sciences, Institutskaya str., 4/1, Novosibirsk 630090, Russia; 4 Department of Physics, Novosibirsk State University, Pirogova str., 2, Novosibirsk 630090, Russia

**Keywords:** Janus-like, nanoparticles, XRD analysis, mechanism of formation, TaSi_2_/Si

## Abstract

Metal-semiconductor Janus-like nanoparticles with the composition tantalum silicide-silicon (TaSi_2_/Si) were synthesized for the first time by means of an evaporation method utilizing a high-power electron beam. The composition of the synthesized particles were characterized using high-resolution transmission electron microscopy (HRTEM), X-ray diffraction (XRD), selective area electron diffraction (SAED), and energy dispersive X-ray fluorescence (EDX) analysis. The system is compared to previously synthesized core-shell type particles in order to show possible differences responsible for the Janus-like structure forming instead of a core-shell architecture. It is proposed that the production of Janus-like as opposed to core-shell or monophase particles occurs due to the ability of Ta and Si to form compounds and the relative content of Ta and Si atoms in the produced vapour. Based on the results, a potential mechanism of formation for the TaSi_2_/Si nanoparticles is discussed.

## 1. Introduction

The unique properties of composite Janus-like (JL) nanoparticles have sparked the interest of researchers in recent years, resulting in a large number of publications about their synthesis (for example [1,2]). JL nanoparticles are particles with two halves in which the two halves of the particle are composed of two different materials. JL nanoparticles can have desirable properties which arise from the way in which the constituent materials are bound and spatially oriented with respect to each other. An example of such a property is the enhanced photocatalytic activity of JL particles composed of photocatalytic materials compared to the photocatalytic activity of the individual constituent materials. This enhancement is thought to stem from the heteroboundry interface between the two materials. This was shown for Janus Au-TiO_2_ particles which possessed enhanced photocatalytic properties in comparison with TiO_2_ or composite Au-TiO_2_ nanoparticles [2]. JL nanoparticles can also possess a large dipole moment; caused by the different nature of the two constituent components [3,4]. A high dipole moment makes it possible to remotely control the position of these nanoparticles by means of electric and magnetic fields with a high spatial resolution [3,4].

Tantalum silicide (TaSi_2_) has an attractive combination of properties, including a high melting point of 2200 °C [5], high thermal stability [6], low electrical contact resistance [6,7], a high modulus of elasticity [8], a high resistance to oxidation in air [6], and a good compatibility with silicon [6,7,8]. Due to its low electrical resistance and oxidation resistance, TaSi_2_ has been utilized in switching devices as Schottky barriers, ohmic contacts, and connectors in integrated circuits [6,9]. In the present work, JL particles with a metallic TaSi_2_ end and a semiconducting Si end were synthesized. This composition should lead to a spatial separation of electrons and the appearance of polarization charges, leading to the presence of a dipole moment. Thus, the spatial orientation of these JL nanoparticles should be controllable by the interaction of the dipole moment with an external electromagnetic field [10].

## 2. Results

**Figure 1:**
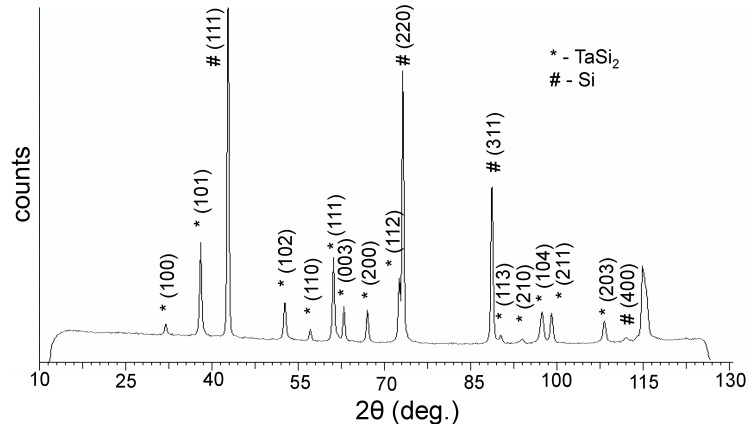
X-ray diffractogram (XRD) of the obtained powder.

**Figure 2:**
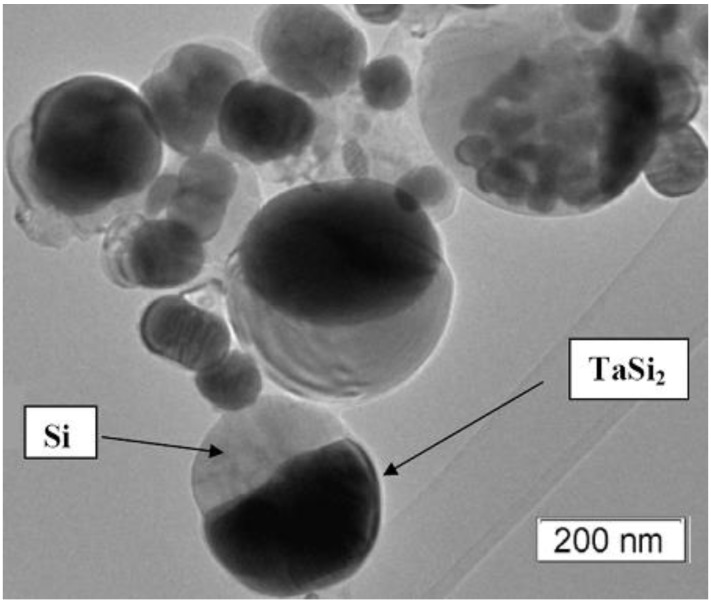
Transmission electron microscopy (TEM) image of the obtained TaSi_2_/Si nanoparticles.

This paper reports the first synthesis of composite JL TaSi_2_/Si nanoparticles. X-ray diffraction (XRD) analysis (Figure 1) showed that the composite powders are composed of two phases: a TaSi_2_ phase [11] and a Si phase [12]; the Si phase being more prominent. Additionally, an XRD of the material which remained in the graphite crucible after evaporation was measured; revealing that it was composed of Ta_5_Si_3_ [13]. Transmission electron microscopy (TEM) images of the obtained particles (Figure 2) show that sphere-like JL particles were obtained which were composed of two parts: a darker coloured half, likely TaSi_2_, and a lighter coloured half, likely silicon; based on the densities of the two species. The obtained powder as a whole contained a mixture of these JL particles as well as monophase particles with the same colour as the lighter half of the JL particles; agreeing with the higher Si content in the powder as measured by XRD. Based on the high-resolution TEM (HRTEM) images, both the darker and lighter phases appeared to be crystalline. However, a thin outer layer (<10 nm) of the lighter phase was amorphous on all observed particles A few particles were observed to have two or three darker regions connected to a lighter phase instead of just one. A few of the darker phases were covered by a thin layer of the lighter phase. The average size of the JL particles was 150 nm ± 13 nm based on over 250 measurements and particle sizes were distributed in a lognormal distribution.

**Figure 3:**
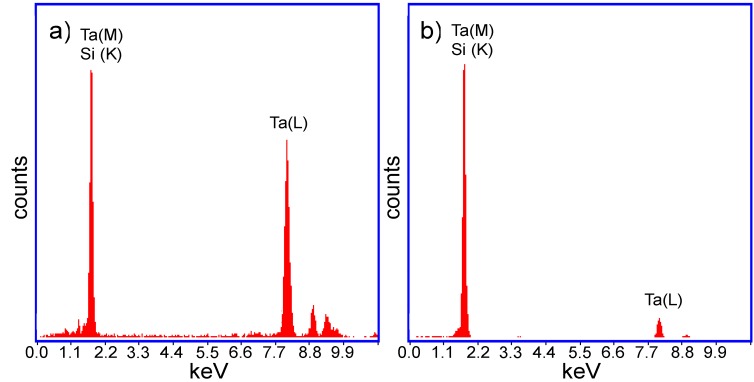
Energy dispersive X-ray fluorescence (EDX) spectra of (**a**) The darker (TaSi_2_) region; and (**b**) The lighter (Si) region of the obtained TaSi_2_/Si JL nanoparticles.

**Figure 4:**
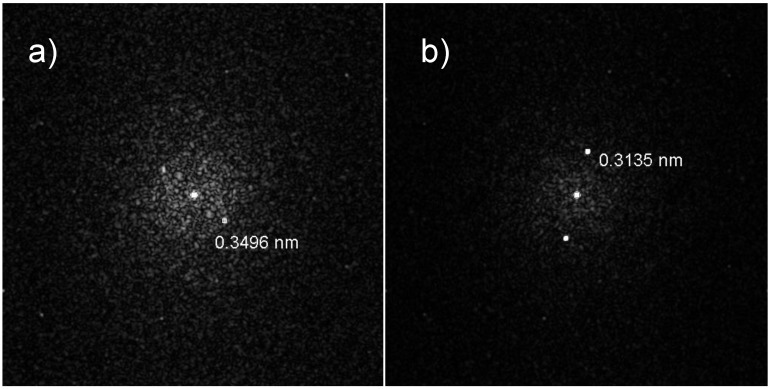
Representative selective area electron diffraction (SAED) diffraction patterns obtained from (**a**) The darker region (TaSi_2_); and (**b**) Lighter region (Si) of the obtained JL nanoparticles.

The hypothesis about the composition of the two halves of the JL particles was confirmed through energy dispersive X-ray fluorescence (EDX) (Figure 3) and selective area electron diffraction (SAED) (Figure 4) analysis on the two halves of the TaSi_2_/Si nanoparticles. In the measured EDX spectra, a strong peak is present around 1.7 keV for both halves of the nanoparticles. As both silicon and tantalum have transitions around 1.7 keV (Kα transition of Si = 1.739 keV, M transition of Ta = 1.709 keV), the absence of Ta in the lighter coloured half of the particle could not be confirmed based on just that peak. However, a strong peak around 8 keV exists on the spectrum of the darker region of the nanoparticle. This corresponds to the Lα transition of Ta (8.145 keV). As this secondary Ta peak is very small on the spectrum of the lighter region of the particle, the EDX measurements support that the darker region is composed of TaSi_2_ while the lighter region is composed of Si. SAED measurements on the dark regions of the nanoparticles measured d-spacings around 3.5 Å which correspond to the interlayer spacing of the (101) planes of TaSi_2_ (3.51 ± 0.15 Å [10]). For the lighter regions, d-spacings of around 3.1 Å which correspond to the (111) planes of Si (3.1356 Å [12]) were also measured. Thus, on the basis of XRD, EDX, and SAED measurements, we can determine that the dark regions of the JL nanoparticles on the TEM images are composed of crystalline TaSi_2_ and the light regions are composed of crystalline Si. SAED measurements also confirmed that the monophasic particles were composed of crystalline silicon.

**Figure 5:**
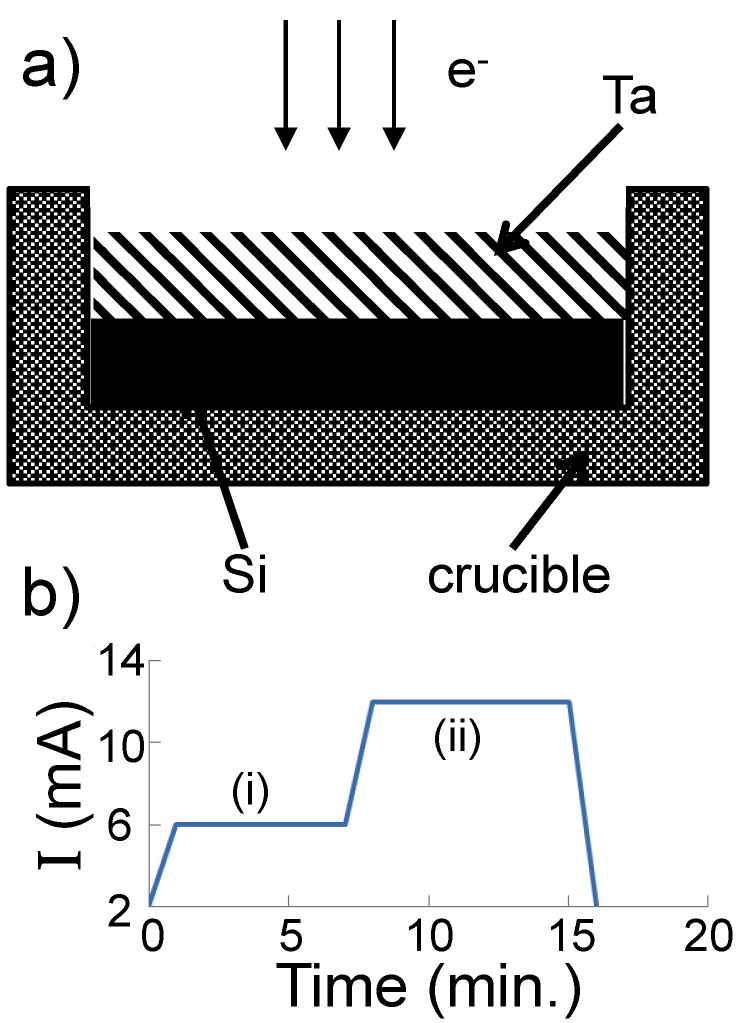
(**a**) Schematic of the Ta and Si arrangement in a graphite crucible before electron beam irradiation; (**b**) Dependence of the electron beam current on time. The materials in the crucible are melted in region (i) and evaporated in region (ii).

In our initial experiments, pieces of Si and Ta were mixed (rather than layered as in the reported experiment) in the graphite crucible prior to evaporation. However, when the precursors were arranged in this fashion, it was found that the products were only Si nanopowders, with no Ta. This indicated that the silicon melted and evaporated before the Ta could melt. As the vapor pressure of Si (*P_v_*(Si) = 4.07 × 10^6^ atm at *T* = 1623 K [14]) is higher than that of Ta (*P_v_*(Ta) = 6.66 × 10^−6^ atm at *T* = 3269 K [15]) and has a much lower melting (*T_m_*(Si) = 1687 K, *T_m_*(Ta) = 3293 K [5]) and boiling (*T_b_*(Si) = 3538 K, *T_b_*(Ta) = 5731 K) point, Si was placed at the bottom of the graphite crucible and Ta on top prior to evaporation in the reported experiment (Figure 5a).

## 3. Discussion

We can speculate that the layered arrangement of materials used in the reported experiment compared to the mixed arrangement used in the initial experiments increased the heating rate of Ta and decreased the heating rate of Si. Thus, between about 1400–2000 °C, at the interface between the Ta and Si, a homogeneous Ta-Si liquid phase is thought to form as it is more thermodynamically favourable for Ta; based on the Ta-Si phase diagram [5]. By the end of the melting phase, the homogeneous Ta-Si liquid will have filled the crucible. During the evaporation phase, the evaporation rates of Ta and Si in the Ta-Si liquid would be expected to be higher and lower, respectively, to some degree compared to pure Ta (*R_exap_*(Ta) = 6.80 × 10^−5^ g·cm^−2^·s^−1^ at *T* = 3269 K [15]) and Si (*R_exap_*(Si) = 14.1 × 10^7^ g·cm^−2^·s^−1^ at *T* = 1623 K [14]). However, it is still expected that the evaporation rate of Si would still be significantly higher than that of Ta and thus the Si concentration in the vapour would be much higher than that of Ta. This is supported by the fact that the material which remained in the crucible after the experimental run was Ta_5_Si_3_ (a Ta-Si compound with less than 10 weight percent silicon) as well as that only monophasic silicon particles but no monophasic tantalum particles were observed in the powder.

A similar arrangement of substances, taking into account the difference in the vapor pressure of the evaporated species, has previously been used by us in the preparation of Cu@Si [16] and Ag@Si [17] core-shell particles by electron beam evaporation. During the evaporation of Ta and Si, similar conditions for the time variation in the electron beam strength, carrier gas flow rate, and geometry of installation were used as during the synthesis of Cu@Si and Ag@Si. Thus, it was surprising that instead of core-shell particles such as Cu@Si and Ag@Si, TaSi_2_/Si JL particles formed for the Ta-Si system. Based on thermodynamic calculations [18], core-shell type nanoparticles tend to form either due to a large difference in the surface tension of the core and shell material (the core-shell configuration achieving the lowest surface energy for the system) or due to a large difference in the atomic sizes of the core and shell material. In the Ta-Si system, the surface tension of Ta (σ(*T*)_Ta_ = {2150 − 0.21(*T* − *T_m_*(Ta))} mN·m^−1^, *T* ≥ *T_m_*(Ta) [19]) is much higher than the surface tension of Si (σ(*T*)_Si_ = {820 − 0.3(*T* − *T_m_*(Si))} mN·m^−1^, *T* ≥ *T_m_*(Si) [20]) while their atomic radii (*r*(Ta) = 146 pm, *r*(Si) = 111 pm) are not significantly different. Thus, purely based on this information, core-shell particles would be expected to form.

However, there are a few differences in the Ta-Si system compared to the Ag-Si and Cu-Si systems. Unlike the Ag-Si and Cu-Si systems, which form no and very few (only within a narrow range of compositions) chemical compounds between the two components, respectively, the Ta-Si system forms a series of compounds (Ta, Ta_3_Si, Ta_2_Si, Ta_5_Si_3_, TaSi_2_, and Si) as can be seen from the Ta-Si phase diagram [5]. Based on the melting temperatures of the Ta-Si phases (*T_m_*(Ta_3_Si) = 2340 °C, *T_m_*(Ta_2_Si) = 2440 °C, *T_m_*(Ta_5_Si_3_) = 2550 °C, *T_m_*(TaSi_2_) = 2040 °C), it can be seen that it is much more thermodynamically favourable for Ta atoms to form into TaSi_2_ particles rather than pure Ta or other Ta-Si phases. This has been shown previously by Ko *et al.* [21] who hot-pressed Ta and Si powders together. The resulting material was found to contain TaSi_2_ and Si grains rather than Ta and Si grains. The expected high silicon ratio compared to tantalum in the evaporated vapour also supports the formation of TaSi_2_ which is the last Ta containing phase to form before pure Si. In comparison, in the Cu-Si system, which can form Cu-Si compounds with lower melting points than Cu or Si, all the Cu-Si compounds require many atoms to form each compound (Cu_38_Si_7_, Cu_9_Si_2_, Cu_15_Si_4_, Cu_19_Si_6_) which makes them less likely to form from the gas phase and more likely that the Cu and Si vapour would nucleate into core-shell particles. Additionally, there is a stronger driving force for Ta to form TaSi_2_ as the difference in the melting temperature of Ta and TaSi_2_ is 980 °C, much larger than the difference in melting temperature between Cu and any of the Cu-Si compounds (around 600 °C).

Thus, it can be speculated that the high surface energy of Ta and the likely excess of Si in the gas makes it more energetically favourable for the Ta atoms to condense into TaSi_2_ rather than pure Ta and Si particles or other Ta-Si compounds. Silicon also prefers to form pure Si particles rather than TaSi_2_ (as it has a lower melting point) and so excess silicon condenses into pure Si particles. Thus, we propose that the TaSi_2_/Si JL nanoparticles form by the following mechanism: Initial nucleation of the gas upon condensation proceeds according to the reaction:

$${Ta}_{\left(g\right)}+4{Si}_{\left(g\right)}\to {TaSi}_{2(s)}+2{Si}_{\left(s\right)}$$

where Ta forms TaSi_2_ with Si and excess Si forms pure Si particles. The two sides of the nucleate are then built up as Ta and Si continue to condense onto the TaSi_2_ side and Si condenses onto the Si side. Although to the authors’ knowledge, there is no empirical data on the surface tension of TaSi_2_, the simple surface tension model proposed by Ergy [22] for bimetallic systems shows that the surface tension of TaSi_2_ will most likely lie between the surface tension values of Ta and Si; closer to Si. Further, the likely excess Si in the binary phase (darker shell half) will rapidly decreases the surface tension from the value of pure TaSi_2_ due to surface segregation effects. Thus, the surface tension difference between the Si and TaSi_2_ halves is likely to not be as great as between Si and Ta and has the possibility of being quite close; thus forming JL particles rather than core-shell type particles. Local fluctuations in the gas composition are expected to account for variations in the size ratios between the TaSi_2_ and Si halves of the JL particles.

XRD analysis of the alloy remaining in the graphite crucible after Si and Ta were evaporated by electron beam irradiation gave a diffraction pattern corresponding to Ta_5_Si_3_. This may be due to the stoichiometry between the Ta and Si in the melt after the evaporation process was stopped and the melt solidified. The stoichiometry of Ta and Si in the melt at the end of the process is likely to be determined by the starting ratio of the two materials (1:1 by weight) and their relative evaporation rates as well as the evaporation time. Thus, if a different amount of each material were placed into the crucible prior to evaporation or different evaporation times were used, different Ta-Si phases or a solid solution of two phases may be left in the crucible at the end of the experiment. Further tests will be required to confirm this.

## 4. Experimental Section

Composite TaSi_2_/Si powders were obtained using an ELV direct-6 electron accelerator (Budker Institute of Nuclear Physics, Novosibirsk, Russia). Schematics of the device and its principles of operation are described in [16,23]. The incident electron energy was 1.4 MeV and the beam current was varied from 3 to 10 mA. In this accelerator, the target material can be irradiated by the electron beam at atmospheric pressure in a gas of the user’s choice (argon in this case), rather than a vacuum. The maximum power density of the electron beam is 106 W/cm^2^. In this work, the following procedure was used for the preparation of TaSi_2_/Si JL particles: A pure monocrystalline silicon ingot was melted by an electron beam into a graphite crucible. Upon termination of the electron beam, the silicon cooled down, solidifying and taking the form of the inside of the crucible. On top of the silicon, pieces of pure tantalum were placed (Figure 5a). The weight ratio of Ta to Si was 1:1. Ta and Si were then heated by the electron beam to their melting temperatures; resulting in a composite liquid. In accordance with the phase diagram of Ta and Si [5], at high temperatures, bulk Ta and Si are mixed in the liquid phase. Next, the beam current is increased to a value at which intense evaporation of the mixed composite liquid occurs. A plot of the electron beam current against time is shown in Figure 5b. The mixed vapors were transferred to a cold zone where they were condensed into nanoparticles by the argon carrier gas. The gas with the particles then passed through a special woven filter onto which the nanoparticles were deposited. The nanopowder sample was then collected from this filter. Previous nanopowder synthesis experiments using the high-powered electron beam evaporation process have produced powders at rates between hundreds of g/h [24] to kg/h [25,26,27] and thus similar production rates are expected for this synthesis. The obtained nanoparticles were characterized using XRD, TEM, HRTEM, SAED, and EDX analysis. These measurements were performed on a JEM-2010 TEM (JEOL, Tokyo, Japan; 200 kV accelerating voltage, 0.14 nm resolution) equipped with an LZ5 EDX spectrometer (Oxford Instruments, Wycombe, UK; 130 eV energy resolution, 1 nm spatial resolution) and a DIFRAY 401 (Scientific Instruments, Saint-Petersburg, Russia) XRD operated using a Cr X-ray source (λ = 2.2897 Å). To prepare the samples for microscopy measurements, the nanopowders were dispersed in ethanol by ultrasonication followed by precipitation of the sample onto a carbon film fixed on a copper grid.

## 5. Conclusions

Janus-like nanoparticles composed of a TaSi_2_ half and a Si half were synthesized by electron beam evaporation of Ta and Si for the first time. The obtained powder contained a mixture of JL particles along with some silicon nanoparticles and silicon nanoparticles with more than one TaSi_2_ phase connected to it. The composition of the JL particles was confirmed through TEM, diffraction, and EDX analysis techniques. JL particles are thought to form from the Ta-Si system instead of core-shell or monophasic particles due to the relative surface energy of Ta and Si, their ability to form into compounds, and the gas composition. Future work will investigate the properties of these TaSi_2_/Si JL particles and their applications, in particular towards electrical applications.
